# Associations of Cytomegalovirus and Muscle Weakness in Older Adults: Results from the Health and Retirement Study

**DOI:** 10.1093/geroni/igaa057.2252

**Published:** 2020-12-16

**Authors:** Kate Duchowny, Grace Noppert

**Affiliations:** 1 University of California, San Francisco, San Francisco, California, United States; 2 University of North Carolina at Chapel Hill, Chapel Hill, North Carolina, United States

## Abstract

Muscle weakness, measured by grip strength, is associated with disability, physical functioning and mortality; however, the sociobiologic underpinnings of muscle weakness are poorly understood. Immune response to cytomegalovirus (CMV), as measured by immunoglobulin G (IgG) antibody levels, is often used as a marker of immunosenescence with higher antibody levels suggesting accelerated immunosenescence. Previous research has shown immune response to CMV across the life course is socially patterned with disadvantaged groups having higher antibody levels across the life course. Thus, the primary objective of this study was to examine associations between CMV IgG antibodies and grip strength in a nationally representative cohort of older adults, and the role of social factors in these associations. Using data from the Health and Retirement Study (N= 4,251; Mean Age= 67.4 years), we employed gender-stratified weighted linear regression models to estimate the mean difference in grip strength associated with tertiles of CMV IgG response controlling for age, race/ethnicity (White, Black and Hispanic) and educational attainment. For men, higher IgG antibody levels were associated with decreased mean grip strength. For example, men in the highest level of IgG antibody had a grip strength of 37.67 kg (95% CI: 36.30, 39.05) compared to men seronegative to CMV with a grip strength of 38.96 kg (95% CI: 38.18, 39.74). For women, there was little difference in grip strength by CMV IgG response. Our findings suggest accelerated immunosenescence may be an important predictor of reduced grip strength in older adults, and critical to understanding social disparities in grip strength.

